# An Unusual Cause of Anaemia of Chronic Disease: Lisinopril-Induced Chronic Inflammatory State

**DOI:** 10.1155/2011/939080

**Published:** 2011-10-26

**Authors:** Toby Eyre, Victoria Van-Hamel-Parsons, Lai Mun Wang, Kathryn A. Hughes, Timothy J. Littlewood

**Affiliations:** ^1^Department of Haematology, Oxford Radcliffe Hospital NHS Trust, Headley Way, Headington, Oxford OX3 9DU, UK; ^2^Department of Cellular Pathology, Oxford Radcliffe Hospital NHS Trust, Headley Way, Headington, Oxford OX3 9DU, UK; ^3^The Manor Surgery, Osler Road, Headington, Oxford OX3 9BP, UK

## Abstract

We report the case of a patient with severe systemic symptoms (weight loss, malaise, and anorexia), eosinophilic oesophagitis, and raised inflammatory markers coinciding with the use of lisinopril. The onset of symptoms occurred after the administration of lisinopril and resolved shortly after cessation of the medication. Despite thorough investigation, no other cause of the systemic inflammation and anaemia of chronic disease was found. “Drug rash with eosinophilia and systemic symptoms” (DRESSs) syndrome describes a potentially serious multiorgan inflammatory response to certain classes of drugs; this includes the use of ACE inhibitors. Although this patient did not meet strict criteria for DRESSs, the subacute inflammatory syndrome with eosinophilic organ infiltration bears similar features. ACE inhibitors should be considered in the differential diagnosis in patients with nonspecific systemic inflammation and anaemia of chronic disease where no other cause is found.

## 1. Introduction

ACE inhibitors are a class of drug known to cause inflammation and allergic reactions. It is well recognised that this class of medication causes inflammatory eosinophilia localised to lung parenchyma [1–3], and the medication is also implicated in DRESSs syndrome (drug rash with eosinophilia and systemic symptoms) [4, 5] and rarely in gastrointestinal eosinophilic infiltration [6].

Systemic inflammation can result in the anaemia of chronic disease through well-studied mechanisms. These include a cytokine-driven inability to utilise reticuloendothelial iron stores and blunting of erythropoietin stimulation. The commonest causes of anaemia of chronic disease are malignancy, chronic infection, and autoimmune disease. Medications, in this case lisinopril, can be implicated in similar pathophysiological processes.

## 2. Case Report

**Figure 1:**
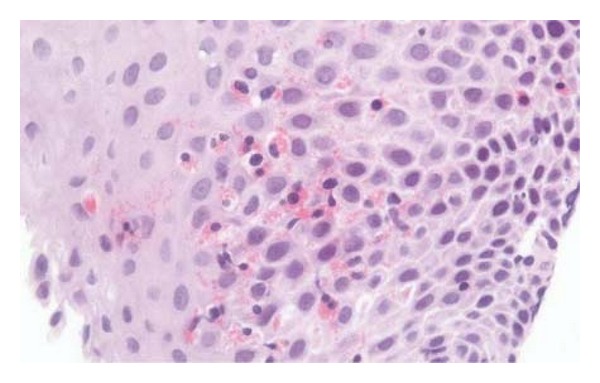
Haematoxylin- and eosin-(H+E) stained slide of oesophageal biopsy. Note the infiltration and clustering of eosinophils, characterized by their bright red cytoplasmic granules.

A 63-year-old lady presented to her GP in February 2010 with dyspepsia. She was otherwise well, but her blood pressure was 150/90. She was prescribed omeprazole 20 mg od for dyspepsia and lisinopril 10 mg od for hypertension. Her haemoglobin was 13.9 g/dL, white cell count 7.37  × 10^9^/L (eosinophil count 0.07  × 10^9^/L), and platelets 303  × 10^9^/L prior to treatment. The lisinopril induced a dry cough, but the medication was continued. Over subsequent months, her gastrooesophageal reflux symptoms worsened and an endoscopy was performed in July 2010. Histology from an oesophageal biopsy showed marked mucosal thickening with prominent intraepithelial eosinophils seen throughout, occurring in aggregates with microabscess formation. There was no evidence of dysplasia or malignancy. A diagnosis of eosinophilic oesophagitis was made (see biopsy image, Figure 1). No specific treatment was given.

By November 2010, she had become unwell with anorexia, weight loss of 7 kg, and generalised malaise. She continued to have dyspepsia. Since February 2010, blood test results revealed the development of a normocytic, normochromic anaemia, a modest eosinophilia and raised inflammatory markers including polyclonal hypergammaglobulinaemia and an increase in the ferritin concentration (Table 1). She was treated with ferrous sulphate with no response. 

She was referred to the haematology department in January 2011 for further investigation of the anaemia and weight loss. A detailed history and physical examination revealed no additional abnormal findings. A chest-abdomen-pelvis (CT) was performed and ruled out an occult malignancy, and there was no evidence of infection. Her liver, renal, thyroid function and bone profile were normal. Her tissue transglutaminase was negative. She reported that she had started to feel unwell after starting the treatment for hypertension, and it was considered whether lisinopril was the explanation for her symptoms and laboratory abnormalities. The drug was stopped at the end of February 2011.

One week after stopping the lisinopril, her systemic and gastrointestinal symptoms resolved. By April 2011, her haemoglobin concentration had increased and the ESR, ferritin, and hypergammaglobulinaemia all started to resolve (Table 1); these continued to improve and she was well in June 2011, with her weight improving towards her initial baseline.

## 3. Discussion

The differential diagnosis of a normochromic, normocytic anaemia with raised inflammatory markers is broad and includes chronic infection, autoimmune disease, and malignancy. A careful history, examination, and appropriate investigations usually reveal the diagnosis.

This patient provided the clue towards her diagnosis when she said that her symptoms started after she commenced treatment for hypertension. This could, of course, have been coincidental, and the hypertension could have been a presenting feature of a systemic disease such as polyarteritis. Evidence that the cause of her symptoms was a systemic inflammatory reaction to lisinopril is circumstantial but strongly supported by the onset and subsequent decline of local (upper gastrointestinal) symptoms, systemic symptoms (malaise, anorexia, weight loss), and the systemic inflammatory response. Since stopping the ACE inhibitor, the anaemia has improved, the ESR and ferritin have normalised, and the polyclonal hypergammaglobulinaemia has resolved. 

ACE inhibitors causing eosinophilic syndromes have been described. As well as causing the commonly known side effect of a cough, ACE inhibitors have been associated with severe pulmonary eosinophilia [1], eosinophilic pneumonia [2], and hypersensitive lung disease [3]. It is noteworthy that our patient developed a cough after the introduction of lisinopril. 

ACE inhibitors are a class of medication implicated in DRESSs “drug rash with eosinophilia and systemic symptoms” syndrome. This syndrome describes an inflammatory reaction associated with a rash, fever, anorexia, lymphadenopathy, single or multiorgan eosinophilic involvement, and peripheral blood hypereosinophilia. This normally occurs 2 to 6 weeks after commencing treatment, and fatalities have been described. Drugs associated with this characteristic syndrome include anticonvulsants, beta-blockers, allopurinol, and sulphonamides [4, 5]. Although our patient did not strictly meet the criteria for this syndrome, similar features including local organ eosinophilic infiltration are described in this subacute, drug-induced, inflammatory process. The exact pathophysiological mechanism for DRESSs syndrome is not fully elucidated, and there is ongoing debate regarding its classification and treatment. 

Eosinophilic oesophagitis has become a well-described phenomenon and is felt to represent an allergic response to an allergen, mediated by T helper 2 cells, IL-5, and IgE [7]. Aero-allergens and food allergens have been implicated [8], as well as two cases observed in carbamazepine-induced hypereosinophilic syndrome [9]. Interestingly, carbamazepine is the most commonly implicated medication in DRESSs syndrome. ACE inhibitors have been rarely associated with gastrointestinal eosinophilic infiltration [6], although not specifically oesophageal infiltration. 

## 4. Concluding Remarks

This patient's health problems, attributed to a lisinopril-induced systemic eosinophilic inflammation, highlight the importance of considering medication side effects in the differential diagnosis of patients with a systemic inflammatory process of unknown aetiology. Peripheral blood eosinophilia, localised organ involvement, and systemic inflammation are well described with the use of ACE inhibitors and should be considered in the differential diagnosis in patients taking an ACE inhibitor and presenting with such features.

## Figures and Tables

**Table 1:** Patient's blood test trends, demonstrating the period for which lisinopril was taken.

		Lisinopril started 02/2010					Lisinopril stopped 02/2011			
	09.02.10	03.11.10	22.11.10	20.12.10	18.01.11	01.02.11	22.02.11	21.03.11	21.04.11	15.06.11

ESR (mm/hr)		73	89	87	90		53	30	30	31
Hb (g/dL)	13.9	10.8	10.6	10.7	10.2	10.4	10.1	10.5	11.3	11.9
Plts (×10^9^/L)	303	362	303	329	289	305	284	252	261	238
MCV (fl)	92.9	89.8	90.1	90.8	91.5	90.9	92.6	95.3	90.4	88.1
RCC (×10^12^/L)	4.76	3.82	3.73	3.82	3.52	3.72	3.63	3.61	4.08	4.2
WCC (×10^9^/L)	7.37	7.78	7.83	7.58	8.04	7.21	6.75	6.34	6.61	6.37
Neut (×10^9^/L)	4.05	4.12	3.76	4.02	4.34	3.32	3.38	2.79	2.84	2.48
Lymph (×10^9^/L)	2.65	2.33	2.66	2.27	2.41	2.67	2.23	2.35	2.58	2.8
Mono (×10^9^/L)	0.59	0.78	0.86	0.83	0.72	0.65	0.61	0.57	0.66	0.64
Eosino (×10^9^/L)	**0.07**	0.54	0.55	0.45	0.48	0.58	0.54	0.63	0.46	0.45
Baso (×10^9^/L)	0.00	0.00	0.00	0.00	0.00	0.00	0.00	0.00	0.00	0
**Ferritin (*μ*g/L)**			**368**	**385**	**525**	**626**	**392**	**303**	**265**	**275**
IgA (g/L)			3.17			3.43	2.73	2.45		2.64
**IgG (g/L)**			**19.2**			**22.2**	**16.5**	**14.8**		**14.9**
IgM(g/L)			1.31			1.38	1.17	1.07		1.16
